# siRNA knockdown validation 101: Incorporating negative controls in antibody research

**DOI:** 10.12688/f1000research.8159.1

**Published:** 2016-03-09

**Authors:** Will Olds, Jason Li

**Affiliations:** 1 Proteintech Group, Rosemont, IL, USA

**Keywords:** knockdown, siRNA, negative control, specificity, Western Blot, shRNA, transfection, vector

## Abstract

More antibody validation protocols would identify non-specific reagents – a major source of irreproducible research – with the inclusion of negative controls. This article presents an overview of one such method: siRNA knockdown. The authors outline a general protocol, the knockdown mechanism, and tips for evaluating knockdown experiments.

## Introduction

Poor quality, unverified antibodies are a major factor underlying the crisis of reproducibility in research ^1^, resulting in an estimated $800 million of waste each year ^2^ and a ten-fold spike in retractions over the past decade ^3^. Behind those numbers are researchers with mounting frustration each time a new batch of reagents fails to reproduce their previous work. If we as a scientific community are to eliminate this problem plaguing our research, we must develop new and more thorough validation methods to fortify and standardize routine studies.

We invite industry to respond to the need for more rigorous validation by incorporating negative controls, an essential part of experimental design that has nonetheless been overlooked historically by antibody manufacturers and distributors. Companies typically validate with Western blots but seldom test routinely whether antibodies still produce a signal when the target protein is suppressed or removed.

**Figure 1.**
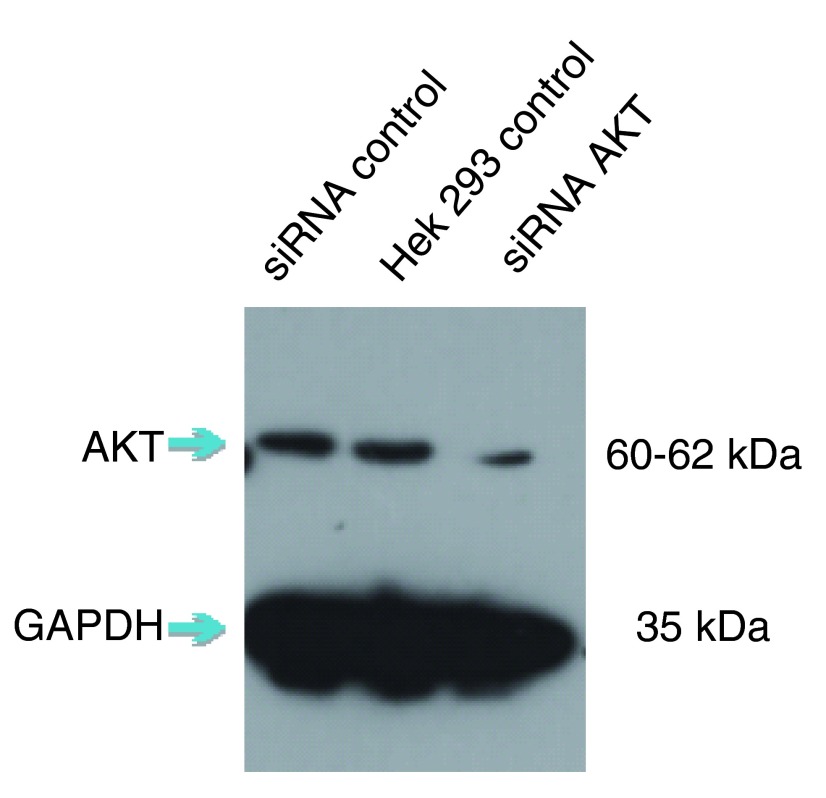
siRNA result of AKT1 antibody (10176-2-AP) with siRNA control, HEK293 control and siRNA AKT1.

Short interfering RNA (siRNA) knockdown is a technology that makes routine use of this strategy more feasible. This technology degrades target messenger RNA to ‘knock down’ the production of a protein in the cell. The combination of siRNA-treated cells and a specific antibody will result in a significant drop in signal compared to an untreated sample by Western blot ( Figure 1).

siRNA knockdown has not been the only validation initiative put forward to identify non-specific antibodies ^4^. CRISPR and other gene editing methods knock out a gene from DNA, preventing the associated protein from ever being produced. By default, an antibody binding to any protein in this environment is binding to the wrong protein. Knock-out is a powerful negative control, however one concern with this method is the risk of cell death when a target protein is vital to cell survival. Other initiatives dispense with negative controls and verify positive identification instead. Mass spectrometry, for example, yields unique spectra that differentiate an antibody-bound protein of interest from any other bound molecule but is limited only to those proteins that can be immunoprecipitated.

While all of these approaches sound simple, their methodologies can be complex. Anyone seeking a deeper understanding of siRNA knockdown validation studies, or considering their own validation protocol, may find the following overview useful. These steps are based on Proteintech methods, which have been tested and refined over years. However, they are by no means the only way to perform this validation.

## Design and engineer a vector

Selecting a target and designing an appropriate vector to transfect into cells is a relatively straightforward process, with an abundance of literature and online resources available to guide the process ( RNAi Consortium, Dharmacon, Ui-Tei, and Genelink). The actual engineering, of course, requires a bit more hands-on finesse to create the short hairpin RNA that is the precursor form of the siRNA. The ultimate aim is to design two single-stranded 19-22mer DNA oligonucleotides (one sense strand, one antisense strand) whose transcription products will eventually anneal together, linked at one end by a short loop sequence. Proteintech uses the loop sequence *TTCAAGACG*.

## Transfect and culture

**Figure 2.**
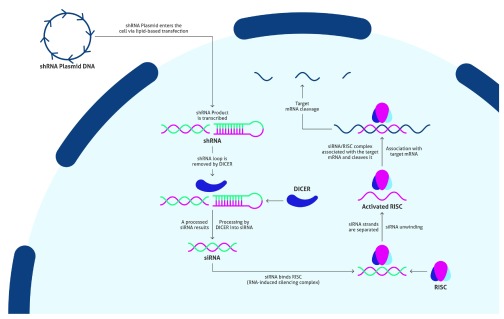
An overview of the biological pathway of siRNA knockdown.

Once the vector has been generated and produced in sufficient quantity, scientists need to determine an appropriate transfection method for delivery into cells. Upon successful transfection, the cells transcribe the foreign DNA to generate the shRNA described above ( Figure 2). Afterward, Dicer processes the shRNA into siRNA by removing the loop sequence. The resulting siRNA binds with RISC (RNA-induced silencing complex), which separates the two strands of the RNA and activates the complex. RISC remains bound to one strand, that complementarily binds to a target mRNA and cleaves it. This suppresses production of the associated protein. Scientists need to allow time for the biological processes to be carried out, while culturing the cells to produce enough sample for a Western blot.

Cell death can be a major source of frustration, particularly if the target protein is vital to cell survival. Fortunately, most cells can propagate normally if the protein is suppressed, but not completely eliminated. In such cases, knockdown methods can be fine tuned and are likely a preferred alternative to knock out validation, which prevents any transcription through gene editing.

## Test and evaluate

A strong signal for the empty-vector cells next to a weak signal for the siRNA-transfected cells in a Western blot means that the antibody is specific and that the knockdown experiment was successful. Any nonspecific bands should raise questions, as these could indicate that the antibodies themselves are nonspecific. Bands should also be consistent across all lanes of the Western blot membrane. If not, something may have gone wrong in the experiment, and protocol and experimental design should be reviewed.

While the value of negative controls to antibody validation protocols should not be understated, it is also understood that such validation is not widely available and that it consumes time and resources to perform in individual labs. Thorough Western blot validation with only positive controls can be a second-best alternative, provided that there is a sufficient quantity of data, and corroborated by published studies that reference the reagent catalog number and manufacturer. We encourage the development and use of negative controls such as siRNA knockdown as the new standard in antibody validation. Doing so will elevate reproducible research and accelerate scientific progress.

## References

[1] Baker M: Reproducibility crisis: Blame it on the antibodies. *Nature.* 2015;521(7552):274–276. 10.1038/521274a 25993940

[2] Baker M: Antibody anarchy: A call to order. *Nature.* 2015;527(7579):545–551. 10.1038/527545a 26607547

[3] McLeod J Ferrigno PK: Antibody Alternatives. *The Scientist.* 2016 Reference Source

[4] Dance A: Exercises for Your Abs. *The Scientist.* 2016 Reference Source

