# Supplementary material for: siRNA knockdown validation 101: Incorporating negative controls in antibody research
Source: F1000Res. 2016 Mar 9;5:308. [Version 1] doi: 10.12688/f1000research.8159.1 (PMC4792204; doi:10.12688/f1000research.8159.1)
Supplement: Peer review history

# Peer review: Referee response for version 1

**DOI:** 10.5256/f1000research.8776.r12834

**Published:** 2016-03-23

**Authors:** Shi-Jiang Lu

**Affiliations:** 1 Ocata Therapeutics Inc, Marlborough, MA, USA

Antibody has been widely used in bio-research fields, and discrepancies among publications using antibodies from different vendors are frequently observed. The main reason is the specificity of antibodies used by different researchers. This article described a siRNA knockdown strategy to validate the specificity of antibodies, which provides a negative control that may serve as an alternative way to exclude non-specific antibodies. This strategy may, at least partially, solve the irreproducible results among researches using antibodies as detecting reagents.

I have read this submission. I believe that I have an appropriate level of expertise to confirm that it is of an acceptable scientific standard.

# Peer review: Referee response for version 1

**DOI:** 10.5256/f1000research.8776.r12835

**Published:** 2016-03-14

**Authors:** Angus Nairn

**Affiliations:** 1 Department of Psychiatry, Yale School of Medicine, New Haven, USA

The suggestion to routinely incorporate negative controls such as siRNA knockdown for the validation of antibodies is a good one. The article is clearly written and timely. The authors might have included an appropriate reference to the discovery of the siRNA knockdown mechanism. Further, the authors might have discussed the basis for choice of cell line used as the positive control.

I have read this submission. I believe that I have an appropriate level of expertise to confirm that it is of an acceptable scientific standard.

# Peer review: Referee response for version 1

**DOI:** 10.5256/f1000research.8776.r12832

**Published:** 2016-03-11

**Authors:** Md. Golam Sharoar

**Affiliations:** 1 Department of Neurosciences, Lerner Research Institute, Cleveland Clinic, Cleveland, OH, USA

The article describes an excellent method of antibody specificity validation before commercial release. Non- specific binding of antibody is a major constrain for reproducibility and for acceptable quality of data. Thus, knocking down the target gene utilizing siRNA is a suitable method to determine the specificity of the antibody. The author explained a suitable method to develop high quality antibody commercialization. I believe that the article is appropriate as published in F1000Research.

I have read this submission. I believe that I have an appropriate level of expertise to confirm that it is of an acceptable scientific standard.
